# Identifying and quantifying metabolites by scoring peaks of GC-MS data

**DOI:** 10.1186/s12859-014-0374-2

**Published:** 2014-12-10

**Authors:** Raphael BM Aggio, Arno Mayor, Sophie Reade, Chris SJ Probert, Katya Ruggiero

**Affiliations:** The University of Auckland, 3A Symonds Street, Auckland, 1142 New Zealand; Department of Gastroenterology, Institute of Translational Medicine, University of Liverpool, Nuffield Building, Crown Street, L693BX Liverpool, UK

**Keywords:** Metabolomics, Identification, GC-MS, Data analysis

## Abstract

**Background:**

Metabolomics is one of most recent omics technologies. It has been applied on fields such as food science, nutrition, drug discovery and systems biology. For this, gas chromatography-mass spectrometry (GC-MS) has been largely applied and many computational tools have been developed to support the analysis of metabolomics data. Among them, AMDIS is perhaps the most used tool for identifying and quantifying metabolites. However, AMDIS generates a high number of false-positives and does not have an interface amenable for high-throughput data analysis. Although additional computational tools have been developed for processing AMDIS results and to perform normalisations and statistical analysis of metabolomics data, there is not yet a single free software or package able to reliably identify and quantify metabolites analysed by GC-MS.

**Results:**

Here we introduce a new algorithm, PScore, able to score peaks according to their likelihood of representing metabolites defined in a mass spectral library. We implemented PScore in a R package called MetaBox and evaluated the applicability and potential of MetaBox by comparing its performance against AMDIS results when analysing volatile organic compounds (VOC) from standard mixtures of metabolites and from female and male mice faecal samples. MetaBox reported lower percentages of false positives and false negatives, and was able to report a higher number of potential biomarkers associated to the metabolism of female and male mice.

**Conclusions:**

Identification and quantification of metabolites is among the most critical and time-consuming steps in GC-MS metabolome analysis. Here we present an algorithm implemented in a R package, which allows users to construct flexible pipelines and analyse metabolomics data in a high-throughput manner.

**Electronic supplementary material:**

The online version of this article (doi:10.1186/s12859-014-0374-2) contains supplementary material, which is available to authorized users.

## Background

Metabolomics, the popular modern approach to screening large numbers of low molecular mass compounds in biological samples, has been successfully applied in drug discovery [1], food science [2] and systems biology [3] studies. The three most commonly used analytical platforms for the identification and quantification of metabolites in biological samples are perhaps gas chromatography-mass spectrometry (GC-MS), nuclear magnetic resonance (NMR) and liquid chromatography-mass spectrometry (LC-MS) [4]. While none of these is stand-alone in the sense that it provides complete coverage of a sample’s metabolome, GC-MS is among the most widely applied because of its ability to separate complex mixtures of metabolites with high efficiency and at low cost [5].

The Automated Mass Spectral Deconvolution System (AMDIS) is the most popular freeware available for metabolite identification and quantification in biological samples analysed by GC-MS [6]. Originally developed for the identification of chemical weapons and related compounds in complex chemical mixtures [7], it is now used in environmental chemistry [8] and metabolomics studies [9]. AMDIS is linked to the NIST standard reference database: one of the most popular mass spectral databases for metabolite identification.

While AMDIS performs well in the identification and quantification of target metabolites within a single biological sample, it does not, in general, use a common reference ion mass fragment (IMF) to quantify the same metabolite across different samples [6]. This limits the reproducibility of the intensity data generated by AMDIS and, therefore, its direct utility for comparative metabolomics studies. Such data may, for example, lead to erroneous identification of chemical signatures (i.e. biomarkers) and, potentially, to the misinterpretation of the activity of metabolic pathways. AMDIS is also known to yield a high rate of false identifications of metabolites, referred to simply as the *false positive rate* [10]. Furthermore, AMDIS reports different results according to the zoom level applied to the chromatogram under analysis. Some compounds are only correctly identified when a smaller portion of the chromatogram is analysed. Finally, the layout of metabolomics data preprocessed by AMDIS is such that it requires further manipulation before it is amenable to subsequent processing and analysis [11]. The necessary manual curation of AMDIS-generated datasets can, therefore, potentially require months to complete.

Recent years have seen exponential growth in the number of metabolomics studies. At the same time, spectral libraries have themselves continued to grow in size, thereby enabling an ever-increasing number of target metabolites to be identified within individual GC-MS-analysed samples. Additionally, high impact scientific journals have raised their standards with respect to the validation of results from metabolomics studies, requiring higher numbers of samples and technical replicates. The net result has been an explosion in the amount of GC-MS-generated data [4], making manual curation post-processing by AMDIS impracticable. An algorithm which more reliably identifies and quantifies metabolites analysed by GC-MS and which is implemented in a software package that reports results in a format that facilitates further data processing without manual intervention is urgently needed.

Numerous programs and software packages to automate processes for the analysis of metabolomics data have become available in the last couple of years. These tools enable quick data normalisation, statistical analysis and the production of graphs for data visualisation [6,12]. Among them is web-based XCMS Online ([13]; https://xcmsonline.scripps.edu/). It is widely used for the comparative analysis (i.e. comparisons between pairs of experimental conditions) of the abundances of *unidentified* IMFs in raw GC-MS data. While XCMS Online enables the identification of metabolites present at significantly different levels across experimental conditions, it is important to note that this involves manual processing. Thus, although XCMS Online can be particularly useful when searching for potential biomarkers, it does not fit the requirements of high-throughput identification and quantification of GC-MS data. Consequently, despite AMDIS’s limitations, it remains the most popular software for the identification and quantification of metabolites in raw GC-MS metabolomics datasets.

We introduce here a new algorithm, PScore, which we have developed for the identification and quantification of metabolites in biological samples analysed by GC-MS. PScore scores the metabolites contained in a pre-defined spectral library according to their likelihood of being associated with a specific chromatographic peak; the higher the score, the greater the similarity between the expected (i.e. defined in the spectral library) and observed spectra and RTs (i.e. measured in the biological sample). For a given metabolite: (1) the closer its fragments’ detected peaks are to its expected RT, (2) the more closely its fragments’ relative intensities follow those defined in the spectral library, and (3) the higher the correlation between the intensities of its fragments, the higher its score. PScore enables the use of threshold scores based on the certainty requirements of each metabolomics experiment, with higher threshold scores resulting in greater precision in compound identification.

PScore is implemented in our new R package, MetaBox, which generates an integrated list of identified metabolites and their corresponding intensities from replicate samples analysed by GC-MS. MetaBox includes functions for removing specific ion mass fragments from GC-MS files and for the generation of graphical outputs. The reports generated by MetaBox can be directly applied to other tools, such as MetaboAnalyst [12] and the R package Metab [6], in order to perform further data processing and statistical analyses. In addition, MetaBox accepts spectral libraries built using AMDIS, including the original formats in which they were generated. Furthermore, MetaBox’s use of pop-up dialog boxes makes it more accessible to novice R users. Finally, being an R package, MetaBox is open-source, allowing users to adapt it to their own pipelines for data analysis.

We validated the results produced by PScore through MetaBox via a two-step approach. First, we compared its performance against AMDIS’s when identifying and quantifying volatile organic compounds (VOCs) present in standard mixtures of metabolites. MetaBox yielded a smaller proportion of misidentifications and higher accuracy in quantification. Second, we used XCMS Online to generate reference datasets for comparing MetaBox’s performance against AMDIS’s when identifying compounds present at different levels in faecal samples from female and male mices. MetaBox yielded a higher percentage of metabolites matching XCMS Online’s results.

## Implementation

### PScore: The algorithm

PScore is a GC-MS-based retention time (RT) scoring algorithm used to assess the likelihood that the observed RTs in a biological sample correspond to known metabolites within a user-defined spectral library.

#### Metabolite identification and quantification by GC-MS

GC-MS instruments usually generate a single file per biological sample, each file containing a list of mass spectra together with their corresponding RTs. These spectra are commonly shown on a chromatogram represented by RT on the horizontal axis and signal intensity on the vertical axis. Peaks in intensity on the chromatogram correspond to putative metabolites in the analysed sample. PScore performs metabolite identification based on a spectral library containing the RT and fragmentation patterns of potential target metabolites.

#### Spectral library requirements

Metabolite identification and quantification require a spectral library containing reference information against which observed spectra can be compared. PScore requires that for each metabolite, *M* say, in a spectral library, *L* say, information is included about its expected retention time, *E*_*RT*_, and typically its four most abundant IMFs’ mass-to-charge (*m*/*z*) ratios, which we will denote by *M*_*i*_ (*i*=1,2,3,4). Additionally, PScore requires that *L* contains the intensity ratios $R_{i}=I_{i'}/I_{1} (i' = 2,3,4)\phantom {\dot {i}\!}$, where $I_{i'}\phantom {\dot {i}\!}$ denotes the expected intensity of IMF $M_{i'}\phantom {\dot {i}\!}$, i.e. *R*_*i*_ is the intensity of $M_{i'}\phantom {\dot {i}\!}$ relative to that of *M*_1_. We will refer to relative intensities simply as intensity *ratios*. For example, consider the first row of the spectral library shown in Table 1, corresponding to the compound ethanol. It has an expected retention time of 6.64 minutes; its four most abundant IMFs have *m*/*z* ratios of 31, 45, 46 and 29; the intensities of the last three of these IMFs, relative to the first, are 0.777, 0.343 and 0.249, respectively.

**Table 1:** **This table shows an example of the mass spectral library required by Pscore, which contains each standard compound’s name (Compound), its expected RT (** ***E***_***RT***_ **) in minutes, the** ***m/z*** **ratio of its four (generally) most IMFs (** ***M***_***1***_ ***,M***_***2***_ ***,M***_***3***_ ***andM***_***4***_ **) and the relative intensities,** ***Ri′*** **, of each** ***Mi′*** **(** ***i*** ^***′***^ ***=2,3,4*** **) to that of** ***M***_***1***_

		**IMF** ***m*** **/** ***z*** ** ratio**					**Intensity relative to** ***M***_**1**_		
**Compound**	***E***_***RT***_	***M***_**1**_	***M***_**2**_	***M***_**3**_	***M***_**4**_		***R***_**2**_	***R***_**3**_	***R***_**4**_
Ethanol	6.64	31	45	46	29		0.777	0.343	0.249
Acetone	7.37	43	58	42	39		0.262	0.076	0.044
Isopropyl alcohol	7.58	45	41	27	39		0.107	0.090	0.072
Acetonitril	7.90	41	40	39	38		0.546	0.223	0.137
Ethyl acetate	10.59	43	45	70	61		0.137	0.116	0.105
1-butanol	13.38	56	41	43	31		0.720	0.543	0.346
2-pentanone	13.95	43	86	41	71		0.249	0.127	0.109
Pyridine	16.42	79	52	51	50		0.564	0.275	0.205
1,2-dimethylbenzene	20.39	91	106	77	51		0.327	0.080	0.077
1,3-dimethylbenzene	20.69	91	106	105	77		0.533	0.223	0.115
1,4-dimethylbenzene	21.80	91	106	105	77		0.488	0.189	0.109
Benzaldehyde	25.71	106	105	77	51		0.990	0.935	0.404
Indole	38.63	117	90	89	63		0.414	0.313	0.103

Many algorithms applied for identifying metabolites analysed by GC-MS, such as AMDIS and X-Rank [14], for example, make use of more than 4 ion mass fragments, if available, when calculating the similarity between two mass spectra. Our experience analysing GC-MS data suggests that the 4 most abundant ion mass fragments and the RT are generally the key factors defining the identity of an analyte. For many compounds, the remaining fragments are generally close to or at the noise level, which increases their variability across samples and may reduce the accuracy in identification. In addition, in the way PScore was developed, every additional fragment to be analysed requires additional computer power, which may considerably increase the analysis’ time. Compounds showing less than 4 fragments in their spectra may have the existent fragments recycled. For example, a compound X containing only the fragments 58 and 106 in their spectra would have these fragments analysed twice by PScore. In this case, the row of the ion library defining compound X would have its most abundant fragment defined as M1 and M3 in the ion library and the second most abundant fragment defined as M2 and M4.

**Figure 1:**
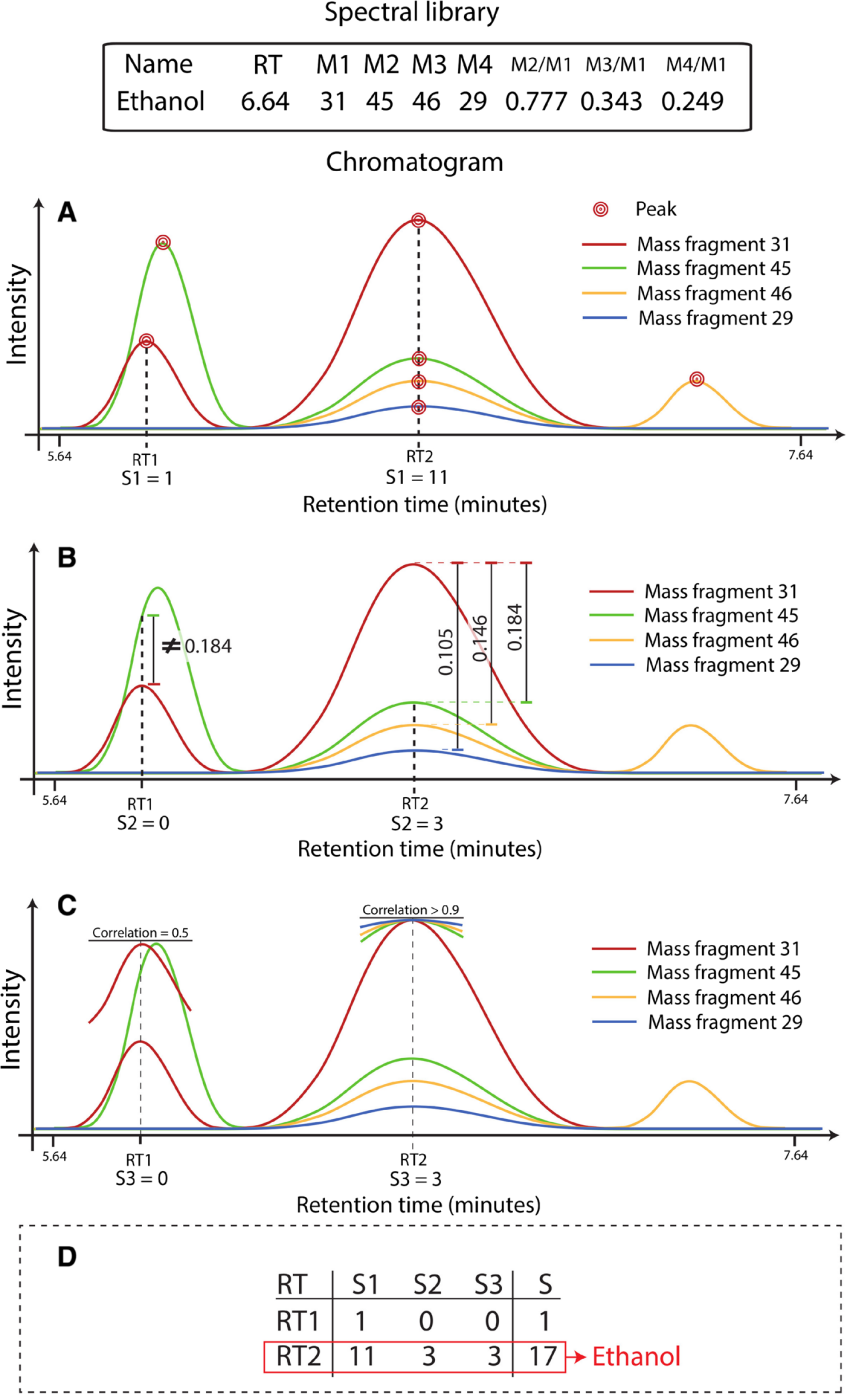
**PScore - algorithm.** PScore searches a GC-MS file for metabolites contained in a defined mass spectral library. It analyses a region of the chromatogram searching for chromatographic peaks representing a metabolite and scores retention times (RT) potentially representing a metabolite if: **(A)** peaks of the IMFs expected to originate from this specific metabolite are present at the same RT and if their intensities are equal to the highest intensity observed for each IMF; if **(B)** these IMFs are detected at the expected proportions defined in the mass spectral library; and **(C)** if the intensities of these IMFs show positive correlation. Finally, **(D)** PScore calculates the final score associated to each potential RT, it assigns the metabolite searched to the RT showing the highest score and registers the intensity of the most abundant mass fragment associated with this metabolite.

In the remainder of this section we describe *PScore*, a peak scoring method which utilises the information available within a single GC-MS sample to score observed peaks occurring within a range of RTs and that are potentially associated with a metabolite, *M*, in the spectral library, *L*. The highest scoring peak is inferred as belonging to *M*. We describe the PScore algorithm according to the four stages shown in Figure 1.

#### Stage 1: Scoring peaks associated with IMFs *M*_1_ – *M*_4_

When a metabolite elutes from the gas chromatography column and enters the mass spectrometer, it is bombarded by electrons and fragmented into ionised components, or IMFs. In theory, the IMFs from the parent metabolite, *M*, should almost simultaneously reach the mass spectrometer’s detector, where their intensities and RTs are recorded. This information is commonly used to build both their individual chromatograms and their cumulative or *total ion chromatogram*. Ideal process would result in entire complement of IMFs yielding a set of overlapping peaks centered precisely on a single expected RT. In practice, however, RT shifts may be observed depending on the type of sample being analysed and the variability across GC-MS runs. Consequently, a metabolite’s IMF peaks may occur in the vicinity of, but not precisely at, its expected RT. Thus, a search must be conducted across a window of RTs spanning the region of the chromatogram which most plausibly contains the IMF peaks corresponding to the metabolite.

Consider a metabolite *M* in spectral library *L* with expected retention time *E*_*RT*_. We define a RT window , with the window parameter, *w*, being user-defined. The region  is searched for groups of peaks potentially corresponding to IMFs *M*_1_,…,*M*_4_ belonging to *M*. The *j*th group’s observed peak intensities are recorded as , where $\hat {I}_{\textit {ij}}$ is the observed intensity of IMF *M*_*i*_ and *t*_*j*_ is the RT at which *M*_1_’s peak is observed. Letting $\hat {I}_{\text {max}}=\text {max}\{\hat {I}_{\textit {ij}}\}$, each observed intensity, $\hat {I}_{\textit {ij}}$, in  is scored according to



The total score for  is the sum over the scores assigned to each of its IMFs, i.e.



allowing a maximum possible score of 12.

#### Stage 2: Similarity scoring of theoretical and observed spectra

If metabolite *M* is present in a GC-MS-analysed sample, not only do we expect a group of peaks to be observed at its expected RT, we also expect its observed intensity ratios to be identical to their corresponding theoretical values in *L*. However, due to variability across GC-MS runs and the possible convolution of metabolites, the values of the observed and theoretical ratios may differ from one another. Thus, at Stage 2 we compute the intensity ratios  from the *j*th group’s observed peak intensities, , where $\hat {R}_{i'j}=\hat {I}_{i'j}/\hat {I}_{1j} (i' = 2,3,4)$. It follows that if the observed intensities in  are from metabolite *M* then we expect $\hat {R}_{i'j}=R_{i'}$ or, equivalently, $\hat {R}_{i'j}/R_{i'}=1$.

We make allowance for variability between observed and theoretical intensity ratios by introducing a *match factorf* (0<*f*<1) which we use to construct intervals around each theoretical ratio, $R_{i'}\phantom {\dot {i}\!}$, associated with metabolite *M*. The lower and upper limits of this interval are given by $L_{i'}={fR}_{i'}\phantom {\dot {i}\!}$ and $U_{i'}=(2-f)R_{i'}\phantom {\dot {i}\!}$, respectively, with the value of *f* chosen to yield sufficiently narrow intervals such that only observed peaks from a group of IMFs corresponding to *M* will lie within them. To reflect this, we give each observed ratio $\hat {R}_{i'j}$ a score of 1 if it falls within its match factor interval $[L_{i'},U_{i'}]\phantom {\dot {i}\!}$. The total score for  is given by the sum over all of its ratios’ scores, i.e.

$$ T_{2j} = \sum_{i'=2}^{4}\mathbf{1}_{\left\{\hat{R}_{i'j}\in [L_{i'},U_{i'}]\right\}}, $$

where

$$\mathbf{1}_{\left\{\hat{R}_{i'j}\in [L_{i'},U_{i'}]\right\}} \left\{\begin{array}{ll} \!\!1, & \text{if}\; \hat{R}_{i'j}\;\in \left[L_{i'},U_{i'}\right] \\ \!\!0, & \text{otherwise} \end{array} \right.. $$

allowing a maximum possible score of 3.

#### Stage 3: Scoring the correlation between IMFs’ intensities

The ion chromatogram of each IMF originated from a single compound is expected to form an approximately bell-shaped curve over a range of RTs *t*_*j*_±*Δ*, where *Δ* is chosen to capture the non-zero intensities with magnitudes that are dependent on RT. We represent this by expressing the intensity of IMF *M*_*i*_ of *M* (*i*>1) as a function of retention time *t*, i.e. $\hat {I}_{\textit {ij}}(t)$. If the IMFs corresponding to the intensities in  are perfectly aligned, then theoretically their intensity ratios would be expected to be constant across *t*∈*t*_*j*_±*Δ*, i.e. $r_{\textit {ij}}(t) = \hat {I}_{\textit {ij}}(t)/\hat {I}_{1j}(t)=c_{\textit {ij}}$, where *c*_*ij*_ denotes the proportionality constant in the linear relationship between $\hat {I}_{\textit {ij}}$ and $\hat {I}_{1j}$ and independent of RT. In other words, IMFs originating from the same compound are expected to have highly correlated intensities, as they are expected to increase and decrease at the same time.

At stage 3 we compute the correlation between the intensities $\hat {I}_{i}$ and *I*_1_, of *M*_1_, (*i*=2,3,4), across the retention time window *t*_*j*_±*Δ*, denoted by $\rho _{i1|t_{j}}$ which is calculated using Pearson’s correlation coefficient. In our experience, the optimal neighborhood of *t*_*j*_ is *Δ*=0.07. Ideally, $\rho _{i1|t_{j}}=1$. However, this is not always the case. Metabolite coelution, for example, may affect the correlation between IMFs’ intensities. Thus, we define a correlation threshold, *ct*, such that 0<*ct*<1. We then give metabolite *M* a score of 1 for each of its observed IMFs at *t*_*j*_ which have $\rho _{i1|t_{j}} \geq ct$; that is, the value of the Pearson’s correlation is greater or equal to the correlation threshold *ct*. The Stage 3 score function is then given by

$$ S_{3j} = \sum_{i=2}^{4} k_{\{ \rho_{i1|t_{j}\pm\Delta} | ct \}}, $$

where

$$k_{\left\{ \rho_{i1|t_{j}\pm\Delta} | ct \right\}} = \left\{ \begin{array}{ll} 1 & \text{if}\; \rho_{i1|t_{j}\pm\Delta} \geq ct \\ 0 & \text{otherwise} \end{array}\right.. $$

Metabolites found at similar RTs, e.g. *RT*_*Ma*_−*RT*_*Mb*_≤|0.1| where *RT*_*Ma*_ is the RT of metabolite *a* and *RT*_*Mb*_ is the RT of metabolite *b*, and sharing IMFs, e.g. ${Ma}_{M_{1}} = {Mb}_{M_{1}}$ where ${Ma}_{M_{1}}$ is the *m*/*z* of IMF *M*_1_ originated from metabolite *Ma* and ${Mb}_{M_{1}}$ is the *m*/*z* of IMF *M*_1_ originated from metabolite *Mb*, may have lower $\rho _{i1|t_{j}}$ and, potentially, lower scoring at stage 3. Three pairwise correlations are scored in Stage 3, which allows a maximum possible score of *S*_3*j*_=3.

#### Stage 4 - Defining the RT and the abundance of metabolite *M*

We calculate the score *S*_*M*_ of metabolite *M* at time *t*_*j*_ by

$$ S_{M_{(t_{j})}} = S_{1_{t_{j}}} + S_{2_{t_{j}}} + S_{3_{t_{j}}}. $$

Then, we obtain the intensity of *M*_1_ at the *t*_*j*_ associated with the highest score, $S_{M_{(t_{j})}}$, and with the lowest difference to the expected RT, *E*_*RT*_. This intensity represents the abundance of *M*_1_.

Stages 1, 2, 3 and 4 are performed for every metabolite *M* in library *L*. After all metabolites in *L* are analysed, it may happen that different metabolites were associated to the same time *t*_*j*_. In these cases, we select for each time *t*_*j*_ only the metabolite showing the highest score $ S_{M_{(t_{j})}} $ and the lowest difference between time *t*_*j*_ and the *E*_*RT*_.

#### Implementing PScore in MetaBox

We have implemented our PScore algorithm in an R package named MetaBox. For each GC-MS sample, it generates a list of metabolites, *M*, with their respective abundances, *P*_*M*(*j*)_, their unique RT, *t*_*j*_, at which they were identified and their calculated score $S_{M_{(t_{j})}}$. MetaBox then merges the results of individual GC-MS samples into a single R data frame called *Total* using metabolite’s names as reference (Additional file 1: Table S1). Optionally, the data frame Total can be exported to a csv file.

Ideally, $S_{M_{(t_{j})}} = 18$ when metabolite *M* is actually present in the analysed sample. However, it is not always the case. A specific compound’s spectrum may vary slightly from sample to sample as a result of GC-MS variation, matrix effect and metabolite coelution. Therefore, we define a score threshold *s*_*t*_, such that 8≤*s*_*t*_≤18. MetaBox then selects metabolites that have a calculated score $S_{M_{(t_{j})}} \ge s_{t}$ and stores them in a second R data frame called *cutOff*, containing the name of each metabolite in the first column and their respective abundances in each GC-MS sample in the following columns (Additional file 1: Table S2). Optionally, the data frame cutOff can be exported to a csv file.

The RT index is an excellent system for obtaining reproducible results within and across labs. It is currently implemented in AMDIS and other tools such as TagFinder [15]. However, PScore was initially developed to use only the RT. The possibility to use the RT index will most probably be implemented in a further version of MetaBox.

## Validation

As we implemented PScore in the R package MetaBox, we compared MetaBox’s performance against AMDIS’s in identifying and quantifying VOCs present in standard mixtures of metabolites and in faecal pellets of female and male mice.

## Methods

### Standard mixtures

A single standard mixture containing 13 metabolites (Table 1) was prepared and divided into 10 aliquots: 5 aliquots of 50 *μ*L and 5 aliquots of 100 *μ*L. Each 50 *μ*L aliquot was diluted by adding 50 *μ*L of water, resulting in a final volume of 100 *μ*L. Each aliquot was then warmed in an incubator oven at 60°C for 30 minutes, then VOCs were adsorbed onto a solid phase microextraction fiber CAR-PDMS 85 *μ*m (Sigma-Aldrich) for 20 minutes and analysed by a Perkin Elmer (Clarus-500) GC-MS using solvent delay, 6 min; temperature program (40°C), 1 min; ramp of 5°C/min to 220°C; finally held at 220°C for 4 min (total run time 41 min). The MS was operated in EI positive mode scanning mass ions in the range 10 to 300 (6–41 min). Room and lab air were used as controls.

#### Metabolite identification

Metabolites were identified using a mass spectral library built using AMDIS and NIST (Version 2.0) (Table 1) (NB. The library used by AMDIS contains additional ions than shown in Table 1). We first characterised algorithm performance on a per-sample basis, calculating the percentage of false positive and false negative metabolite identifications, defining the percentage of false positives as $100p^{\scriptscriptstyle +}_{i}\%$, where $p^{\scriptscriptstyle +}_{i}$ is the proportion of misidentified metabolites (in relation to the total number of identified compounds) in the *i*th standard sample, and the percentage of false negatives as $100p^{\scriptscriptstyle -}_{i}\%$, where $p^{\scriptscriptstyle -}_{i}$ is the proportion of unidentified metabolites in the *i*th standard sample. For example, consider the standard sample described above containing 13 metabolites. If an algorithm identifies 100 metabolites, including 10 of which are in the standard sample, it is reported as having 23.1% of false negatives (i.e. 100×3/13) and 90% of false positives (i.e. 100×90/100).

High percentages of both false positives and false negatives may lead to erroneous inferences being drawn from the data. Optimal metabolite identification tool is one which yields the smallest percentages of both false positives and false negatives. We evaluate the performances of AMDIS and MetaBox over all *n*=10 with these criteria in mind.

The match factor used by AMDIS may affect the number of false negatives and positives reported. Therefore, AMDIS was applied using the match factor values of 70, 80 and 90. MetaBox was applied using match factor of 70, correlation of 0.95 and score cut of 13.

#### Metabolite quantification

All aliquots from the standard mixture were analysed by both AMDIS and MetaBox. For AMDIS, its ‘Base Peak’ values were reported for the metabolite intensities. A reference dataset (Reference), containing the intensity of each metabolite’s most abundant IMF, was manually obtained for each sample using the R package XCMS [16]. The abundances reported by MetaBox, AMDIS and Reference for each metabolite are expected to be very similar. We confirmed this by performing a hierarchical cluster analysis (HCA) and a principal component analysis (PCA) on the combined datasets.

### Mice samples

Five female and five male five-week old inbred wild-type C57BL/6 mice were purchased from Charles River Laboratories (Margate, UK) and acclimated to standard animal house conditions at the University of Liverpool for a minimum of 1 week. The mice were individually housed for a total of 8 weeks, when one ten-pellet faecal sample was taken from a clean cage. Mice were then sacrificed under Schedule 1 Animals Act 1986. Mice were used in accordance with local ethics approved from the University of Liverpool. Each (*n*=10; Female = 5; Male = 5) ten-pellet sample was then analysed by GC-MS using the same configuration described in *Standard mixtures*. The mice samples were analysed using AMDIS and MetaBox, using a mass spectral library built using AMDIS and NIST database (Version 2.0) (Additional file 1: Table S3). In order to remove potential false positives, we only analysed those metabolites present in at least 2 samples per experimental condition (i.e. Female and Male).

It is difficult to generate a reference or control when analysing mice samples, as the identity and concentrations of metabolites in these samples are unknown. Therefore, we applied an approach used for biomarker discovery [16]. We used XCMS Online to generate a reference dataset containing the list of IMFs present at significantly different levels between female and male samples (Welch *t*-test; *p*-value <0.05), including the RT where the peak of each IMF is detected. Then, we used our spectral library (Additional file 1: Table S3), which contains the expected RT and the IMFs of each metabolite, to identify the IMFs reported by XCMS Online. We then conducted a Welch’s *t*-test on the AMDIS and MetaBox datasets comparing males and females for each listed metabolite and compared these algorithms’ performances against the *t*-test results from XCMS Online. For clarity, compounds found at significantly different levels between female and male mice samples will be called as biomarkers. (NB. All chromatograms were left untreated and no data normalisations were applied to metabolite abundances.)

The CAS numbers of all metabolites used in this study are available in Table S7 of the Additional file 1.

## Results and discussion

### Standard mixtures

For clarity, aliquots of 50 *μ*L of standard mixture + 50 *μ*L of water will be described simply as 50 *μ*L samples, while aliquots of 100 *μ*L will be described as 100 *μ*L samples.

#### Metabolite identification

**Figure 2:**
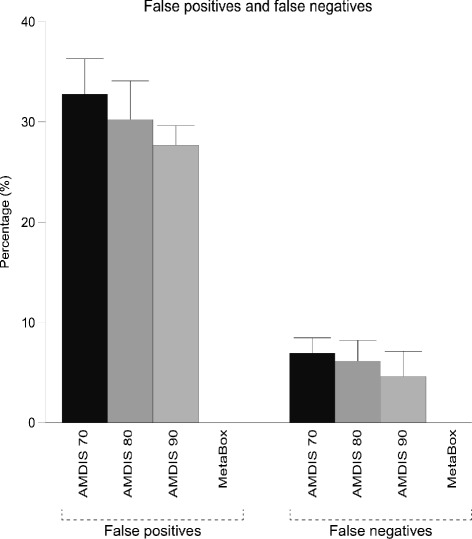
**Average percentages of false positives and false negatives.** A standard mixture containing 13 metabolites was divided in 10 aliquots and analysed by GC-MS. Each sample was then processed by MetaBox and AMDIS using match factors of 70, 80 and 90. Shown are the average percentages, plus error bars representing two times the standard error, of false positives and false negatives produced by each tool. False positives are compounds that are misidentified, while false negatives are unidentified compounds that are present in the standard mixtures.

To enable the comparison of AMDIS’s and MetaBox’s efficacies in metabolite identification, we calculated the percentages of false positives and false negatives reported by each algorithm when analysing 10 samples of a standard mixture of metabolites (i.e. 5 samples of 50 *μ*L and 5 of 100 *μ*L), using match factors of *f*=70,80 and 90 for AMDIS; and match factor of *f*=70 and score cut of 13 for MetaBox. Every compound reported by AMDIS was considered in the analysis, including multiple identifications for a single RT. For *f*=70, AMDIS reported an average ± SE (*n*=10) of 32.8% ± 1.8% of false positives and an average of 6.9% ± 0.8% of false negatives. *f*=80 and 90 resulted in 30.3% ± 1.9% and 27.8% ± 1.0% of false positives, respectively, and 6.2% ± 1.0% and 4.6% ± 1.3% of false negatives, respectively (Figure 2). MetaBox performed overwhelming better than AMDIS, reporting no false positives and no false negatives.

Although, AMDIS performed reasonably well in terms of low percentages of false negatives, it was a poor performer with respect to its high reporting of false positives. It may be that AMDIS is actually performing as expected given the primary motivation for its development, single-sample analyses of complex chemical mixtures to identify any signs of potential target compounds or chemical weapons [7]. In this context a low false negative rate is crucial and AMDIS’s performance meets this requirement. However, the primary motivation for most metabolomics experiments, is the identification and quantification of the highest possible number of metabolites present in biological samples for the comparisons of their abundances, or relative abundances, across experimental conditions. It is non-targeted analysis generally limited only by the metabolites represented in the spectral library. The biological interpretation is then achieved based on the metabolite profile generated by each sample. In this case, the percentages of both false negatives and false positives are crucial for biologically meaningful interpretations of the data. A high percentage of false negatives represents potential losses of biological evidence, while a high percentage of false positives may provide misleading evidences. Therefore, results generated by AMDIS should be manually curated and critically assessed in order to achieve sound biological interpretations.

#### Metabolite quantification

**Figure 3:**
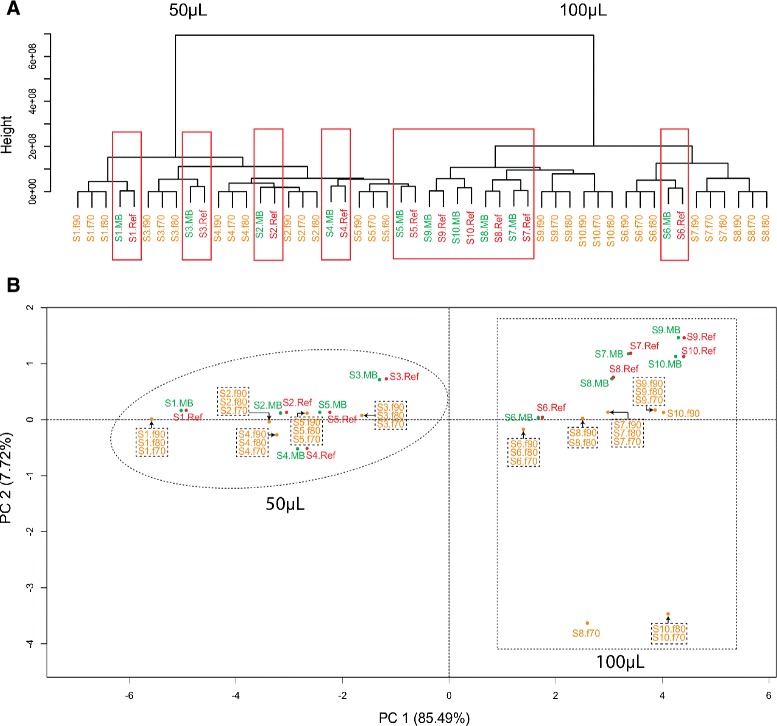
**Hierarchical cluster analysis (HCA) and principal component analysis (PCA).** **(A)** Dendrogram from HCA (euclidean distance; average linkage) and **(B)** scatterplot of first two principal components from PCA on data resulting from the application of AMDIS and MetaBox to the raw data from 10 GC-MS-analysed standard mixture samples (5 × 50 *μ*L+50 *μ*L water and 5 × 100 *μ*L aliquots). Reference datasets (Control) were obtained using the R package XCMS. Samples are labeled using a combination of sample number (e.g. S1 = sample 1) and the algorithm applied (MB = MetaBox, Ref = reference, f# = AMDIS using match factor #=70, 80 or 90).

Average-linkage hierarchical cluster analysis (HCA) (Figure 3A) and principal component analysis (PCA) (Figure 3B) were performed on the metabolite abundances reported by AMDIS and MetaBox (Additional file 1: Table S4). The HCA yielded two main nodes, or clusters: one containing the 50 *μ*L samples and the other the 100 *μ*L samples. Within samples, the MetaBox and reference datasets always clustered together under the same node in the first agglomeration round and this node excluded the corresponding AMDIS dataset. This is indicative of MetaBox-generated abundances being closer in value to those in the reference datasets than the AMDIS-generated ones. The PCA yielded results consistent with those from the HCA, i.e. the 50 *μ*L samples clustered together around negative values of the first principal component (PC 1) while the 100 *μ*L samples clustered around positive values of PC 1. The 50 *μ*L samples varied little in the direction of the second principal component (PC 2), indicating that AMDIS and MetaBox yielded datasets that were similar to one another and to the reference datasets. Samples corresponding to MetaBox-based datasets were always adjacent to the matching reference dataset, showing once again the high degree of agreement between the MetaBox and reference datasets. The 100 *μ*L samples showed separation of datasets in the direction of PC 2. The reference and MetaBox datasets derived from the same sample consistently yielded approximately equal values for PC 2, once again showing a high degree of similarity between the two sets of data. AMDIS, on the other hand, yielded datasets with PC 2 values less than or equal to zero, demonstrating that only when a high match factor is used will AMDIS yield datasets containing abundances approaching values close to those in the reference datasets.

Part of the dissimilarity between the AMDIS and the reference datasets may be a result of background noise subtraction performed by AMDIS and/or the use of different IMFs when deconvoluting and quantifying the same metabolite across samples. The potential use of different IMFs for metabolite quantification by AMDIS is another indication of its development without a view to comparing the same metabolite across different samples, and yet this is a fundamental concern of metabolomics studies. Further evidence lies in the format it uses for reporting results. AMDIS can generate two types of reports: individual reports or a single report (batch report) for several samples by simply appending results sample-by-sample without actually matching metabolites identified in the different samples. Furthermore, AMDIS reports multiple potential identities associated to a single RT. Consequently, when applied to metabolomics studies, AMDIS’s results must be manually cleaned (i.e. the correct hit for each RT must be manually selected), the ion mass fragment used to quantify each metabolite must be manually verified and the results produced for different GC-MS files must be combined in a single table or spreadsheet, and this can be enormously time-consuming depending on the number of samples being processed. MetaBox, however, was developed specially for metabolomics studies. Its results are reported in a single spreadsheet containing the identified metabolites and their respective abundances in every analysed sample, and in the format most commonly required for downstream data normalisation and analysis.

### Mice samples

To compare the efficacies of AMDIS and MetaBox in identifying potential biomarkers, we evaluated the datasets generated by each against the XCMS Online reference dataset. XCMS Online reported a total of 387 IMFs (features), from which 73 showed significantly different intensities (Welch *t*-test; *p*-value <0.05) between female and male mice faecal samples (Additional file 1). Based on the IMFs and RTs in the spectral library used by AMDIS and MetaBox (Additional file 1: Table S3), we identified 19 compounds associated to the total list (387) of IMFs reported by XCMS Online. Eleven compounds were associated to 47 of the 73 IMFs reported by XCMS Online at significantly different intensities between female and male samples (Additional file 1: Table S5). However, only 4 of these compounds (Table 2) showed IMFs that were both present at significantly different levels according to XCMS Online results and used by AMDIS and MetaBox for metabolite quantification. Therefore, only these 4 compounds were expected to be found as potential biomarkers by AMDIS and MetaBox. AMDIS and MetaBox were able to identify all 19 compounds associated to the XCMS Online results (Additional file 1: Table S6). For all match factors tested, AMDIS identified 3 potential biomarkers, being only one confirmed by XCMS Online (Additional file 1: Table S5). MetaBox identified 4 potential biomarkers, being two confirmed by XCMS Online (Additional file 1: Table S5). In summary, AMDIS was able to report 1 out of 4 potential biomarkers, while MetaBox reported 2 out of 4. Although MetaBox missed the identification of 2 potential biomarkers, its results represent 100% improvement in relation to AMDIS’.

**Table 2:** **List of compounds identified from XCMS Online results as differentially abundant (based on Welch** ***t*** **-test) between GC-MS-analysed female (n = 5) and male (n = 5) mice faecal samples**

**Compound**	**MetaBox**	**AMDIS70**	**AMDIS80**	**AMDIS90**
Benzene*	0.186	0.123	0.123	0.123
Hexanal	*0.003*	0.203	0.203	0.366
Pentanal	*0.012*	0.146	0.189	NA
Propanoic acid	0.077	*0.038*	*0.038*	*0.038*

AMDIS analyses were performed using match factors of 70, 80 and 90. *P*-values < 0.05 in italics indicate differentially abundant metabolites.

## Conclusions

Identification and quantification of metabolites is among the most critical and time-consuming steps in GC-MS metabolome analysis. The reliability of the biological inferences that can be drawn from metabolomics studies is directly related to the quality of the data upon which they are based. In addition, as the size and number of metabolomics studies conducted by individual laboratories has grown, the time available to analyse each single dataset has reduced. Therefore, to satisfy the criteria of metabolomics studies ideally software must reliably identify and quantify metabolites, and the results must be reported in a format that facilitates further data analysis. Although AMDIS has been widely used in metabolomics, results show that its performance no longer meets the requirements of modern high-throughput analysis of metabolomics experiments.

We presented here a new algorithm, PScore, which uses a spectral library to analyse GC-MS samples and score retention times according to their probability of representing a metabolite. We implemented PScore in an R package, MetaBox, and compared its performance against AMDIS when analysing standard mixtures of metabolites and mice faecal samples. PScore greatly reduces the percentage of false positives and false negatives, and it considerably improves the quantification of metabolites analysed by GC-MS. In addition, our new R package MetaBox incorporates functions to generate graphical outputs and reports results in a format accepted by other software, such as Metab and MetaboAnalyst, allowing users to perform further data processing and statistical analyses in a high-throughput way. As an R package, MetaBox allows users to construct flexible pipelines for data analysis and allows pop-up dialog boxes, which facilitate its usage by R beginners.

## Availability and requirements

**Project name:** MetaBox**Project home page:**http://raphaelaggio.github.io/**Operating system:** Platform independent**Programing language:** R [17] version 3.0.0 or higher**Other requirements:** R packages xcms [16], svDialogs [18], pander [19] and MassSpecWavelet [20]**License:** General Public License version 3

## Additional files

Additional file 1
**Supplementary data.** File containing the tables to be used as supplementary data.

Additional file 2
**XCMS Online results.** File containing the results from the XCMS Online analysis performed on mice samples.
