# Supplementary material for: Identifying and quantifying metabolites by scoring peaks of GC-MS data
Source: BMC Bioinformatics. 2014 Dec 10;15(1):374. doi: 10.1186/s12859-014-0374-2 (PMC4307155; doi:10.1186/s12859-014-0374-2)
Supplement: Additional file 1 — Supplementary data. File containing the tables to be used as supplementary data. [file 12859_2014_374_MOESM1_ESM.pdf]

Supplementary material - Identifying and quantifying metabolites by scoring peaks of GC-MS data -

Aggio, R.B.M.; Mayor, A.; Reade, S.;
Probert, C.S.J. and Ruggiero, K..

Gastroenterology department
University of Liverpool
Liverpool, UK

The University of Auckland
Auckland, NZ

April 19, 2014

Table 1. Complete report generated by MetaBox - *Total*. The complete report generated by MetaBox contains the retention times where metabolites were identified in each GC-MS sample, their differences between expected (i.e. the ERT in the mass spectral library) and observed retention times (i.e. measured retention time), their scores asigned by MetaBox and their intensities or abundances based on the IMF M1 defined in the mass spectral library used.

Sample	1-butanol	2-pentanone	Acetone	Acetonitril	Benzaldehyde	Ethanol	Ethyl acetate
RT_Sample1_1	13.39	13.96	7.38	7.91	25.72	6.65	10.60
DiffRT_Sample1_1	0.00	0.00	0.00	0.00	0.01	0.00	0.00
Score_Sample1_1	18	18	14	18	18	15	18
Sample1_1	166100992	358105088	248266752	89587712	534921216	23259136	345210880
RT_Sample2_1	13.39	13.96	7.38	7.91	25.71	6.65	10.60
DiffRT_Sample2_1	0.01	0.00	0.00	0.00	0.01	0.00	0.00
Score_Sample2_1	18	18	18	15	15	16	18
Sample2_1	174522368	408633344	285147136	96366592	603521024	24012800	446169088
RT_Sample3_1	13.39	13.96	7.38	7.91	25.71	6.66	10.60
DiffRT_Sample3_1	0.01	0.00	0.00	0.01	0.01	0.01	0.00
Score_Sample3_1	18	15	15	18	15	18	17
Sample3_1	181108736	419954688	271532032	94244864	589234176	24418304	427343872
RT_Sample4_1	13.38	13.96	7.37	7.91	25.71	6.65	10.60
DiffRT_Sample4_1	0.01	0.01	0.00	0.00	0.01	0.00	0.00
Score_Sample4_1	17	18	18	14	18	18	18
Sample4_1	194707456	457605120	307740672	107720704	654049280	24311808	505266176
RT_Sample5_1	13.38	13.95	7.37	7.90	25.70	6.65	10.60
DiffRT_Sample5_1	0.01	0.01	0.00	0.01	0.00	0.00	0.00
Score_Sample5_1	18	17	18	18	18	18	15
Sample5_1	208617472	456081408	315424768	104652800	649789440	25080832	478183424
RT_Sample1_2	13.40	13.98	7.38	7.91	25.68	6.65	10.60
DiffRT_Sample1_2	0.01	0.01	0.00	0.01	0.03	0.00	0.00
Score_Sample1_2	18	18	18	15	15	14	18
Sample1_2	35991552	202309632	140296192	44122112	148930560	11761664	201703424
RT_Sample2_2	13.39	13.97	7.38	7.91	25.69	6.65	10.60
DiffRT_Sample2_2	0.00	0.00	0.00	0.00	0.01	0.00	0.00
Score_Sample2_2	18	16	18	17	16	15	17
Sample2_2	51728384	231981056	183844864	60628992	163987456	13713408	242745344
RT_Sample3_2	13.38	13.96	7.37	7.90	25.70	6.65	10.60
DiffRT_Sample3_2	0.01	0.00	0.00	0.01	0.01	0.00	0.00
Score_Sample3_2	18	18	18	16	14	18	15
Sample3_2	76136448	291504128	211861504	65150976	207470592	15432704	316309504
RT_Sample4_2	13.39	13.98	7.38	7.91	25.71	6.65	10.61
DiffRT_Sample4_2	0.01	0.01	0.00	0.00	0.01	0.00	0.01
Score_Sample4_2	17	16	16	16	14	18	18
Sample4_2	71401472	248872960	194805760	65810432	164790272	15376384	235896832
RT_Sample5_2	13.39	13.97	7.38	7.91	25.69	6.65	10.60
DiffRT_Sample5_2	0.00	0.00	0.00	0.00	0.01	0.00	0.00
Score_Sample5_2	18	18	18	15	14	18	18
Sample5_2	70148096	264830976	193216512	63635456	165134336	14892032	275120128

* Table continues in the next page.

Continuation of Table 1.

Sample	Indole	Isopropyl alcohol	Pyridine	Xylene1	Xylene2	Xylene3
RT_Sample1_1	38.64	7.59	16.44	20.40	20.70	21.81
DiffRT_Sample1_1	0.00	0.00	0.02	0.00	0.00	0.01
Score_Sample1_1	17	18	18	18	17	16
Sample1_1	157777920	81797120	756023296	29389824	86278144	44974080
RT_Sample2_1	38.65	7.60	16.44	20.39	20.70	21.80
DiffRT_Sample2_1	0.01	0.01	0.01	0.00	0.00	0.00
Score_Sample2_1	16	18	16	18	18	18
Sample2_1	165347328	79134720	869531648	53858304	138510336	82784256
RT_Sample3_1	38.63	7.59	16.44	20.40	20.70	21.81
DiffRT_Sample3_1	0.01	0.01	0.01	0.00	0.00	0.01
Score_Sample3_1	15	14	14	18	18	15
Sample3_1	181731328	82141184	843120640	41267200	118095872	65318912
RT_Sample4_1	38.65	7.58	16.43	20.39	20.70	21.80
DiffRT_Sample4_1	0.01	0.01	0.00	0.00	0.00	0.00
Score_Sample4_1	17	18	15	18	16	18
Sample4_1	165494784	91922432	928841728	56934400	164823040	98222080
RT_Sample5_1	38.63	7.58	16.43	20.39	20.68	21.79
DiffRT_Sample5_1	0.01	0.01	0.00	0.00	0.02	0.01
Score_Sample5_1	16	18	14	18	15	16
Sample5_1	183287808	94564352	889716736	54325248	146456576	80572416
RT_Sample1_2	38.64	7.59	16.42	20.40	20.70	21.81
DiffRT_Sample1_2	0.00	0.01	0.01	0.00	0.01	0.00
Score_Sample1_2	18	14	15	16	18	18
Sample1_2	70967296	41697280	369508352	25606144	75735040	44204032
RT_Sample2_2	38.64	7.59	16.43	20.40	20.70	21.81
DiffRT_Sample2_2	0.00	0.00	0.00	0.00	0.01	0.01
Score_Sample2_2	17	15	14	18	15	17
Sample2_2	80273408	53235712	397950976	27309056	79917056	43536384
RT_Sample3_2	38.64	7.59	16.41	20.39	20.70	21.80
DiffRT_Sample3_2	0.00	0.00	0.01	0.00	0.00	0.00
Score_Sample3_2	18	14	15	14	16	18
Sample3_2	84336640	60440576	482574336	35299328	97837056	62398464
RT_Sample4_2	38.65	7.59	16.43	20.41	20.71	21.81
DiffRT_Sample4_2	0.01	0.00	0.00	0.01	0.01	0.01
Score_Sample4_2	18	17	18	17	18	14
Sample4_2	84062208	65531904	438960128	20614144	56647680	32196608
RT_Sample5_2	38.64	7.59	16.42	20.39	20.70	21.81
DiffRT_Sample5_2	0.00	0.00	0.01	0.00	0.00	0.00
Score_Sample5_2	18	14	18	14	18	16
Sample5_2	84557824	59486208	427327488	25833472	76070912	45645824

Table 2. Compacted report generated by MetaBox - $cutOff$**.** The compacted report generated by MetaBox contains the metabolites identified in each GC-MS sample and their respective intensity or abundance based on the IMF M1 in the mass spectral library used.

Sample	Replicates	Ethanol	Acetone	Isopropyl alcohol	Acetonitril	Ethyl acetate	1-butanol
Sample1_1	uL100	23259136	248266752	81797120	89587712	345210880	166100992
Sample2_1	uL100	24012800	285147136	79134720	96366592	446169088	174522368
Sample3_1	uL100	24418304	271532032	82141184	94244864	427343872	181108736
Sample4_1	uL100	24311808	307740672	91922432	107720704	505266176	194707456
Sample5_1	uL100	25080832	315424768	94564352	104652800	478183424	208617472
Sample1_2	uL50	11761664	140296192	41697280	44122112	201703424	35991552
Sample2_2	uL50	13713408	183844864	53235712	60628992	242745344	51728384
Sample3_2	uL50	15432704	211861504	60440576	65150976	316309504	76136448
Sample4_2	uL50	15376384	194805760	65531904	65810432	235896832	71401472
Sample5_2	uL50	14892032	193216512	59486208	63635456	275120128	70148096

Sample	2-pentanone	Pyridine	Xylene1	Xylene2	Xylene3	Benzaldehyde	Indole
Sample1_1	358105088	756023296	29389824	86278144	44974080	534921216	157777920
Sample2_1	408633344	869531648	53858304	138510336	82784256	603521024	165347328
Sample3_1	419954688	843120640	41267200	118095872	65318912	589234176	181731328
Sample4_1	457605120	928841728	56934400	164823040	98222080	654049280	165494784
Sample5_1	456081408	889716736	54325248	146456576	80572416	649789440	183287808
Sample1_2	202309632	369508352	25606144	75735040	44204032	148930560	70967296
Sample2_2	231981056	397950976	27309056	79917056	43536384	163987456	80273408
Sample3_2	291504128	482574336	35299328	97837056	62398464	207470592	84336640
Sample4_2	248872960	438960128	20614144	56647680	32196608	164790272	84062208
Sample5_2	264830976	427327488	25833472	76070912	45645824	165134336	84557824

Table 3. Mass spectral library used to analyse female and male mice faecal samples. A mass spectral library was built in AMDIS using NIST database (Version 2.0) in order to identify compounds present in female and male mice faecal samples analysed by GC-MS. The mass spectral library used by MetaBox contains only the 4 most abundant ions present in the mass spectral library created using AMDIS and NIST.

Compound	ERT	M1	M2	M3	M4	R2	R3	R4
Trimethylamine	6.178	58	59	42	30	0.43	0.406	0.204
Ethanol	6.644	31	45	46	29	0.777	0.343	0.249
Acetone	7.373	43	58	42	39	0.262	0.076	0.044
Isopropyl alcohol	7.582	45	41	27	39	0.107	0.09	0.072
Acetonitril	7.905	41	40	39	38	0.546	0.223	0.137
2-methylpropanal	9.02	43	41	72	27	0.804	0.541	0.375
2,3-butanedione	10.279	43	86	42	15	0.215	0.09	0.075
2-butanone	10.557	43	72	29	27	0.189	0.129	0.091
Ethyl acetate	10.593	43	45	70	61	0.137	0.116	0.105
Acetic acid	12.518	43	45	60	42	0.882	0.622	0.175
1-butanol	13.381	56	41	43	31	0.72	0.543	0.346
2-pentanone	13.959	43	86	41	71	0.249	0.127	0.109
Pentanal	14.224	44	43	29	57	0.778	0.704	0.516
3-hydroxy-2-butanone	16.052	45	43	88	27	0.607	0.147	0.085
Propanoic acid	16.221	74	28	29	73	0.97	0.764	0.679
Pyridine	16.426	79	52	51	50	0.564	0.275	0.205
Hexanal	18.346	43	41	44	56	0.832	0.632	0.544
Butanoic acid	19.597	60	73	41	42	0.412	0.243	0.233
1,2-dimethylbenzene	20.395	91	106	77	51	0.327	0.08	0.077
1,3-dimethylbenzene	20.697	91	106	105	77	0.533	0.223	0.115
3-methyl butanoic acid	21.542	60	43	41	87	0.451	0.392	0.269
2-methyl butanoic acid	21.802	74	41	57	60	0.561	0.425	0.387
1,4-dimethylbenzene	21.803	91	106	105	77	0.488	0.189	0.109
Pentanoic acid	23.068	60	73	41	27	0.447	0.264	0.215
Benzaldehyde	25.712	106	105	77	51	0.99	0.935	0.404
benzene, 1, 3-bis(1,1-dimethylethyl)-	33.807	175	57	190	176	0.322	0.204	0.139
Indole	38.634	117	90	89	63	0.414	0.313	0.103

Table 4. AMDIS, MetaBox and Reference results for samples from the standard mixture of metabolites. A standard mixture containing 13 metabolites was divided in 10 aliquots (5 × 50μL+50μL water and 5 × 100μL aliquots) and analysed by GC-MS. Each sample was then processed by MetaBox and AMDIS using match factors of 70, 80 and 90. Reference datasets (Control) were obtained using the R package XCMS.

Sample	DataClass	Tool	1-butanol	2-pentanone	Acetone	Acetonitril	Benzaldehyde	Ethanol	Ethyl acetate
Sample1_1	100uL	Control	169279488	369115136	248266752	89587712	534921216	23438336	345210880
Sample2_1	100uL	Control	176668672	412483584	285147136	96366592	603521024	24012800	446169088
Sample3_1	100uL	Control	181108736	419954688	271532032	95674368	589234176	24418304	427343872
Sample4_1	100uL	Control	194707456	457605120	307740672	108470272	654049280	25887744	505266176
Sample5_1	100uL	Control	208617472	456081408	315424768	107765760	649789440	26106880	495058944
Sample1_2	50uL	Control	35991552	202309632	141000704	47382528	148930560	11761664	201703424
Sample2_2	50uL	Control	51818496	234209280	183975936	60628992	174538752	13939712	247562240
Sample3_2	50uL	Control	76873728	314114048	211894272	65150976	207470592	15432704	316309504
Sample4_2	50uL	Control	71401472	248872960	202162176	65810432	172556288	15524864	252280832
Sample5_2	50uL	Control	72912896	264830976	207060992	64122880	173506560	14892032	275120128
Sample1_1	100uL	AMDIS-70	162705504	338734112	248266752	86069256	507925344	23116038	342671360
Sample2_1	100uL	AMDIS-70	176572160	390337664	273528480	92706168	553533504	23244864	428855712
Sample3_1	100uL	AMDIS-70	175314240	373280448	265655360	88899080	562246464	23021846	415200384
Sample4_1	100uL	AMDIS-70	192901888	371490432	299235168	101441680	626002112	25464128	477256704
Sample5_1	100uL	AMDIS-70	199360496	435956000	299254656	103398208	617978944	25233962	482676704
Sample1_2	50uL	AMDIS-70	34300908	192980720	141000704	43608332	139180208	11607327	197190784
Sample2_2	50uL	AMDIS-70	50734364	223163936	177081840	57517692	163987456	13701553	247269568
Sample3_2	50uL	AMDIS-70	76873728	294932832	204587376	62216292	196911408	14863259	304683712
Sample4_2	50uL	AMDIS-70	68043072	219055216	190834848	62993084	163550112	15240865	243230544
Sample5_2	50uL	AMDIS-70	69858384	257555104	196959424	60626644	161729264	14152269	272799360
Sample1_1	100uL	AMDIS-80	162705504	338734112	248266752	86069256	507925344	23116038	342671360
Sample2_1	100uL	AMDIS-80	176572160	390337664	273528480	92706168	553533504	23244864	428855712
Sample3_1	100uL	AMDIS-80	175314240	373280448	265655360	88899080	562246464	23021846	415200384
Sample4_1	100uL	AMDIS-80	192901888	371490432	299235168	101441680	626002112	25464128	477256704
Sample5_1	100uL	AMDIS-80	199360496	435956000	299254656	103398208	617978944	25233962	482676704
Sample1_2	50uL	AMDIS-80	34300908	192980720	141000704	43608332	139180208	11607327	197190784
Sample2_2	50uL	AMDIS-80	50734364	223163936	177081840	57517692	163987456	13701553	247269568
Sample3_2	50uL	AMDIS-80	76873728	294932832	204587376	62216292	196911408	14863259	304683712
Sample4_2	50uL	AMDIS-80	68043072	219055216	190834848	62993084	163550112	15240865	243230544
Sample5_2	50uL	AMDIS-80	69858384	257555104	196959424	60626644	161729264	14152269	272799360
Sample1_1	100uL	AMDIS-90	162705504	338734112	248266752	86069256	507925344	23116038	342671360
Sample2_1	100uL	AMDIS-90	176572160	390337664	273528480	92706168	553533504	23244864	428855712
Sample3_1	100uL	AMDIS-90	175314240	373280448	265655360	88899080	562246464	23021846	415200384
Sample4_1	100uL	AMDIS-90	192901888	371490432	299235168	101441680	626002112	25464128	477256704
Sample5_1	100uL	AMDIS-90	199360496	435956000	299254656	103398208	617978944	25233962	482676704
Sample1_2	50uL	AMDIS-90	34300908	192980720	141000704	43608332	139180208	11607327	197190784
Sample2_2	50uL	AMDIS-90	50734364	223163936	177081840	57517692	163987456	13701553	247269568
Sample3_2	50uL	AMDIS-90	76873728	294932832	204587376	62216292	196911408	14863259	304683712
Sample4_2	50uL	AMDIS-90	68043072	219055216	190834848	62993084	163550112	15240865	243230544
Sample5_2	50uL	AMDIS-90	69858384	257555104	196959424	60626644	161729264	14152269	272799360
Sample1_1	100uL	MetaBox	166100992	358105088	248266752	89587712	534921216	23259136	345210880
Sample2_1	100uL	MetaBox	174522368	408633344	285147136	96366592	603521024	24012800	446169088
Sample3_1	100uL	MetaBox	181108736	419954688	271532032	94244864	589234176	24418304	427343872
Sample4_1	100uL	MetaBox	194707456	457605120	307740672	107720704	654049280	24311808	505266176
Sample5_1	100uL	MetaBox	208617472	456081408	315424768	104652800	649789440	25080832	478183424
Sample1_2	50uL	MetaBox	35991552	202309632	140296192	44122112	148930560	11761664	201703424
Sample2_2	50uL	MetaBox	51728384	231981056	183844864	60628992	163987456	13713408	242745344
Sample3_2	50uL	MetaBox	76136448	291504128	211861504	65150976	207470592	15432704	316309504
Sample4_2	50uL	MetaBox	71401472	248872960	194805760	65810432	164790272	15376384	235896832
Sample5_2	50uL	MetaBox	70148096	264830976	193216512	63635456	165134336	14892032	275120128

* Table continues in the next page.

Continuation of Table 4.

Sample	DataClass	Tool	Indole	Isopropyl alcohol	Pyridine	Xylene1	Xylene2	Xylene3
Sample1_1	100uL	Control	157777920	82120704	766640128	29983744	86278144	44974080
Sample2_1	100uL	Control	167673856	79134720	869531648	53858304	141459456	82890752
Sample3_1	100uL	Control	181731328	82141184	843120640	41664512	118910976	66351104
Sample4_1	100uL	Control	167837696	93126656	928841728	57958400	164823040	98222080
Sample5_1	100uL	Control	186777600	95952896	889716736	55349248	146456576	80572416
Sample1_2	50uL	Control	70967296	41697280	369508352	25606144	76808192	44204032
Sample2_2	50uL	Control	80273408	53235712	415711232	27378688	79917056	43917312
Sample3_2	50uL	Control	86499328	61800448	482574336	35684352	100077568	62398464
Sample4_2	50uL	Control	84062208	65531904	454852608	20614144	57167872	32196608
Sample5_2	50uL	Control	84557824	60612608	444088320	25833472	76689408	45645824
Sample1_1	100uL	AMDIS-70	151852176	78793672	731381760	28611962	83735200	NA
Sample2_1	100uL	AMDIS-70	166854656	74937064	812839040	53321184	129915520	NA
Sample3_1	100uL	AMDIS-70	153310896	82141184	768369024	40554172	117930968	NA
Sample4_1	100uL	AMDIS-70	162741856	88016912	906598080	56565344	158983856	NA
Sample5_1	100uL	AMDIS-70	180838960	90944912	878686080	54127808	140060032	NA
Sample1_2	50uL	AMDIS-70	70967296	31689264	330948544	24909594	72615568	NA
Sample2_2	50uL	AMDIS-70	78669880	50478668	410904384	26507924	78379776	NA
Sample3_2	50uL	AMDIS-70	82398632	58627184	435843424	34661036	96895912	NA
Sample4_2	50uL	AMDIS-70	81107400	62048104	409744320	20033856	56077676	NA
Sample5_2	50uL	AMDIS-70	84180784	57760808	404575680	25833472	73178088	45218040
Sample1_1	100uL	AMDIS-80	151852176	78793672	731381760	28611962	83735200	NA
Sample2_1	100uL	AMDIS-80	166854656	74937064	812839040	53321184	129915520	NA
Sample3_1	100uL	AMDIS-80	153310896	82141184	768369024	40554172	117930968	NA
Sample4_1	100uL	AMDIS-80	162741856	88016912	906598080	56565344	158983856	NA
Sample5_1	100uL	AMDIS-80	180838960	90944912	878686080	54127808	140060032	2762552
Sample1_2	50uL	AMDIS-80	70967296	31689264	330948544	24909594	72615568	NA
Sample2_2	50uL	AMDIS-80	78669880	50478668	410904384	26507924	78379776	NA
Sample3_2	50uL	AMDIS-80	82398632	58627184	435843424	34661036	96895912	NA
Sample4_2	50uL	AMDIS-80	81107400	62048104	409744320	20033856	56077676	NA
Sample5_2	50uL	AMDIS-80	84180784	57760808	404575680	25833472	73178088	45218040
Sample1_1	100uL	AMDIS-90	151852176	78793672	731381760	28611962	83735200	NA
Sample2_1	100uL	AMDIS-90	166854656	74937064	812839040	53321184	129915520	NA
Sample3_1	100uL	AMDIS-90	153310896	82141184	768369024	40554172	117930968	NA
Sample4_1	100uL	AMDIS-90	162741856	88016912	906598080	56565344	158983856	NA
Sample5_1	100uL	AMDIS-90	180838960	90944912	878686080	54127808	140060032	NA
Sample1_2	50uL	AMDIS-90	70967296	31689264	330948544	24909594	72615568	NA
Sample2_2	50uL	AMDIS-90	78669880	50478668	410904384	26507924	78379776	NA
Sample3_2	50uL	AMDIS-90	82398632	58627184	435843424	34661036	96895912	NA
Sample4_2	50uL	AMDIS-90	81107400	62048104	409744320	20033856	56077676	NA
Sample5_2	50uL	AMDIS-90	84180784	57760808	404575680	25833472	73178088	45218040
Sample1_1	100uL	MetaBox	157777920	81797120	756023296	29389824	86278144	44974080
Sample2_1	100uL	MetaBox	165347328	79134720	869531648	53858304	138510336	82784256
Sample3_1	100uL	MetaBox	181731328	82141184	843120640	41267200	118095872	65318912
Sample4_1	100uL	MetaBox	165494784	91922432	928841728	56934400	164823040	98222080
Sample5_1	100uL	MetaBox	183287808	94564352	889716736	54325248	146456576	80572416
Sample1_2	50uL	MetaBox	70967296	41697280	369508352	25606144	75735040	44204032
Sample2_2	50uL	MetaBox	80273408	53235712	397950976	27309056	79917056	43536384
Sample3_2	50uL	MetaBox	84336640	60440576	482574336	35299328	97837056	62398464
Sample4_2	50uL	MetaBox	84062208	65531904	438960128	20614144	56647680	32196608
Sample5_2	50uL	MetaBox	84557824	59486208	427327488	25833472	76070912	45645824

Table 5. XCMS Online results and potential biomarkers. Ten (5 × Female and 5 × male) mice faecal samples were analysed by XCMS Online, MetaBox and AMDIS using the match factors 70, 80 and 90. Spectral Library indicates the IMFs used by MetaBox and AMDIS for metabolite quantification (N.B. The library used by AMDIS contains additional ions). XCMS indicates the IMFs reported by XCMS Online at significantly different levels between female and male mice faecal samples (Welch t-test; p-value < 0.05). ID indicates if IMFs reported by XCMS Online were positively identified based on the similarity (± 0.2 min) between the RTs reported by XCMS Online and the RTs in the mass spectral library used by AMDIS and MetaBox; and on the IMFs reported by XCMS Online and the IMFs present in the mass spectral library. $p - values$ indicates the results of Welch t-tests on the results produced by MetaBox and AMDIS using match factors 70, 80 and 90. Underlined compounds indicate potential biomarkers, based on the fact that they present IMFs both reported by XCMS Online at significantly different levels between female and male mice faecal samples and are part of the mass spectral library used by AMDIS and MetaBox. IMFs in bold were both reported by XCMS Online at significantly different levels and are part of the mass spectral library used by AMDIS and MetaBox.

	Sapectral Library						$p - values$			
Compound	M1	M2	M3	M4	XCMS	ID	MetaBox	AMDIS-70	AMDIS-70	AMDIS-90
1-butanol	56	41	43	31	None	No	NA	NA	NA	NA
2-butanone	43	72	29	27	29,61	Yes	PSEC	0.039	0.039	0.039
2-methyl butanoic acid	74	41	57	60	None	Yes	0.788	0.750	0.750	0.750
2-methylpropanal	43	41	72	27	None	Yes	0.704	0.711	0.711	0.711
2-pentanone	43	86	41	71	None	Yes	0.494	0.610	0.610	0.610
2,3-butanedione	43	86	42	15	None	Yes	0.636	0.691	0.691	0.691
3-hydroxy-2-butanone	45	43	88	27	16	Yes	0.908	0.956	0.956	0.956
3-methyl butanoic acid	60	43	41	87	None	Yes	0.979	0.954	0.954	0.954
Acetic acid	43	45	60	42	14,15,19	Yes	0.214	0.173	0.173	0.173
Acetone	43	58	42	39	None	Yes	0.965	0.937	0.937	0.937
Acetonitril	41	40	39	38	None	No	0.049	0.299	0.299	0.218
Benzaldehyde	106	105	77	51	None	Yes	0.797	0.272	0.272	0.272
Benzene*	175	**57**	190	176	29,39,51,**57**	Yes	0.186	0.123	0.123	0.123
Butanoic acid	60	73	41	42	25	Yes	0.507	0.495	0.495	0.495
Ethanol	31	45	46	29	15,19,42	Yes	0.166	0.171	0.171	0.171
Ethyl acetate	43	45	70	61	None	No	NA	NA	NA	NA
Hexanal	**43**	41	44	56	**	Yes	0.040	0.203	0.203	0.366
Indole	117	90	89	63	None	No	0.724	0.505	0.505	0.577
Isopropyl alcohol	45	41	27	39	14,19,38,40,55	Yes	0.063	0.058	0.058	NA
Pentanal	**44**	43	**29**	**57**	***	Yes	0.000	0.146	0.189	PSEC
Pentanoic acid	60	73	41	27	26,39	Yes	0.237	0.234	0.234	0.234
Propanoic acid	74	**28**	29	73	25,**28**,55,56,75	Yes	0.077	0.038	0.038	0.038
Pyridine	79	52	51	50	None	No	NA	NA	NA	NA
Trimethylamine	58	59	42	30	None	No	NA	NA	NA	NA
1,2-dimethylbenzene	91	106	77	51	None	Yes	0.019	0.254	0.019	0.019
1,3-dimethylbenzene	91	106	105	77	None	No	0.478	0.022	0.229	NA
1,4-dimethylbenzene	91	106	105	77	None	No	NA	NA	NA	NA

* Benzene denotes 1,3-bis(1,1-dimethylethyl)benzene
** IMFs: 15,26,29,30,38,39,40,**43**,51,53,54,67
*** IMFs: 26,27,**29**,39,41,42,**44**,55,**57**
PSEC = Present in a Single Experimental Condition

Table 6. MetaBox and AMDIS results for mice samples. Ten faecal samples from female (n=5) and male (n=5) mices were processed by GC-MS and further analysed by MetaBox and AMDIS using mach factors of 70, 80 and 90.

	Mean Female (n = 5)				Mean Male (n = 5)			
Name	MetaBox	AMDIS70	AMDIS80	AMDIS90	MetaBox	AMDIS70	AMDIS80	AMDIS90
1-butanol	NA	396939	396939	396939	NA	1130986	1130986	1130986
2-butanone	NA	27560024	27560024	27560024	69828608	41334846	41334846	41334846
2-methyl butanoic acid	6933862	6632403	6632403	6632403	7915520	7753413	7753413	7753413
2-methylpropanal	6492467	5977665	5977665	5977665	6063514	5584594	5584594	5584594
2-pentanone	17666253	16526651	16526651	16526651	20007731	18211596	18211596	18211596
2,3-butanedione	175953510	169922330	169922330	169922330	154847642	152707122	152707122	152707122
3-hydroxy-2-butanone	514277376	502115240	502115240	502115240	498096538	494710682	494710682	494710682
3-methyl butanoic acid	15773235	15210433	15210433	15210433	15997235	15675630	15675630	15675630
Acetic acid	629139046	610652557	610652557	610652557	747461018	751325210	751325210	751325210
Acetone	226155725	220868008	220868008	220868008	223002624	215322234	215322234	215322234
Acetonitril	2596208	1937014	1937014	1937014	3538227	2283186	2283186	2460764
Benzaldehyde	1324438	2228126	2228126	2228126	1237491	1460219	1460219	1460219
Benzene*	6844570	6467469	6467469	6467469	9959117	9986088	9986088	9986088
Butanoic acid	229801984	222438170	222438170	222438170	262086656	254716197	254716197	254716197
Ethanol	32213197	31327672	31327672	31327672	40442266	39513648	39513648	39513648
Ethyl acetate	NA	NA	NA	NA	NA	2460764	2460764	2460764
Hexanal	15499008	15612779	15612779	17595507	30657195	25318087	25318087	26004199
Indole	1194646	1390284	1390284	1390284	1392906	1781638	1781638	1945388
Isopropyl alcohol	262678118	244841838	244841838	316431584	503562240	489230883	489230883	218592592
Pentanal	4894549	5969086	6186450	5505856	11208192	8625962	9150959	9408886
Pentanoic acid	4938342	4667730	4667730	4667730	9226035	9053801	9053801	9053801
Propanoic acid	83587891	82326229	82326229	82326229	123643494	127618013	127618013	127618013
Pyridine	NA	NA	NA	NA	NA	18211596	18211596	18211596
Trimethylamine	NA	NA	NA	NA	43053056	21696374	21696374	21696374
Unknown 1	19932160	19380422	19380422	19380422	22819226	22115467	22115467	22115467
Unknown 2	NA	NA	NA	NA	5222016	5015608	5015608	6495946
1,2-dimethylbenzene	2372390	2058813	2600574	2600574	1406259	1548102	1548102	1548102
1,3-dimethylbenzene	711398	627578	708718	732413	811664	2270900	2239141	1661767
1,4-dimethylbenzene	330885	NA	NA	NA	421040	NA	NA	NA

* Benzene denotes 1,3-bis(1,1-dimethylethyl)benzene

Continuation of Table 6.

	$t-Test$			
Name	MetaBox	AMDIS70	AMDIS80	AMDIS90
1-butanol	NA	NA	NA	NA
2-butanone	PSEC	0.039	0.039	0.039
2-methyl butanoic acid	0.788	0.750	0.750	0.750
2-methylpropanal	0.704	0.711	0.711	0.711
2-pentanone	0.494	0.610	0.610	0.610
2,3-butanedione	0.636	0.691	0.691	0.691
3-hydroxy-2-butanone	0.908	0.956	0.956	0.956
3-methyl butanoic acid	0.979	0.954	0.954	0.954
Acetic acid	0.214	0.173	0.173	0.173
Acetone	0.965	0.937	0.937	0.937
Acetonitril	0.049	0.299	0.299	0.218
Benzaldehyde	0.797	0.272	0.272	0.272
Benzene*	0.186	0.123	0.123	0.123
Butanoic acid	0.507	0.495	0.495	0.495
Ethanol	0.166	0.171	0.171	0.171
Ethyl acetate	NA	NA	NA	NA
Hexanal	0.040	0.203	0.203	0.366
Indole	0.724	0.505	0.505	0.577
Isopropyl alcohol	0.063	0.058	0.058	NA
Pentanal	0.000	0.146	0.189	PSEC
Pentanoic acid	0.237	0.234	0.234	0.234
Propanoic acid	0.077	0.038	0.038	0.038
Pyridine	NA	NA	NA	NA
Trimethylamine	NA	NA	NA	NA
Unknown 1	0.254	0.256	0.256	0.256
Unknown 2	NA	NA	NA	NA
1,2-dimethylbenzene	0.019	0.254	0.019	0.019
1,3-dimethylbenzene	0.478	0.022	0.229	NA
1,4-dimethylbenzene	NA	NA	NA	NA

* Benzene denotes 1,3-bis(1,1-dimethylethyl)benzene
PSEC = Present in a Single Experimental Condition

Table 7. Metabolites' CAS numbers. The CAS numbers of all metabolites analysed in this study were collected from the NIST database (Version 2.0).

Compound	CAS Number
Trimethylamine	75-50-3
Ethanol	64-17-5
Acetone	67-64-1
Isopropyl alcohol	67-63-0
Acetonitril	75-05-8
2-methylpropanal	78-84-2
2,3-butanedione	431-03-8
2-butanone	78-93-3
Ethyl acetate	141-78-6
Acetic acid	64-19-7
1-butanol	71-36-3
2-pentanone	107-87-9
Pentanal	110-62-3
3-hydroxy-2-butanone	513-86-0
Propanoic acid	79-09-4
Pyridine	110-86-1
Hexanal	66-25-1
Butanoic acid	107-92-6
1,2-dimethylbenzene (Xylene1)	95-47-6
1,3-dimethylbenzene (Xylene2)	108-38-3
1,4-dimethylbenzene (Xylene3)	106-42-3
3-methyl butanoic acid	503-74-2
2-methyl butanoic acid	116-53-0
Pentanoic acid	109-52-4
Benzaldehyde	100-52-7
1,3-bis(1,1-dimethylethyl)benzene	1014-60-4
Indole	120-72-9
